# Mental Health Response to Disasters: Is There a Role for a Primary Care-Based Clinician?

**DOI:** 10.1017/S1049023X22001194

**Published:** 2022-10

**Authors:** David Crompton, Jane Shakespeare-Finch, Gerard FitzGerald, Peter Kohleis, Ross Young

**Affiliations:** 1. Queensland University of Technology, Brisbane, Queensland, Australia; 2. Griffith University, Queensland, Australia; 3. Metro South Hospital and Health Service, Woolloongabba, Queensland, Australia; 4. University Sunshine Coast, Maroochydore DC, Queensland, Australia

**Keywords:** community, health, mental, response, rural

## Abstract

**Introduction:**

Following natural disasters, rural general practitioners (GPs) are expected to undertake several roles, including identifying those experiencing psychological distress and providing evidence-informed mental health care. This paper reports on a collaborative mental health program developed to support a rural GP practice (population <1,500) and a disaster response service.

**Methods:**

The program provided specialized disaster mental health care via the placement of a clinician in the GP facility. In collaboration with the GP practice, the program offered opportunistic screening using the Primary Care Posttraumatic Stress Disorder (PTSD) Scale (PC-PTSD) for probable PTSD as the primary measure and the Kessler 6 (K6) as a secondary measure. Those scoring higher than two on the PC-PTSD scale were referred to the mental health clinician (MHC) for further assessment and treatment.

**Results:**

Sixty screening assessments were completed. Fourteen patients (male = 3; female = 11) scored higher than two on the PC-PTSD. The referred group PC-PTSD mean score was 3.14 and K6 mean score of 19. Those not referred had a PC-PTSD mean score = 0.72 and K6 mean score = 7.30. The treatment and non-treatment groups differed significantly (PC-PTSD: P <.00001 and K6: P <.00001). A prior history of trauma exposure was notable in the intervention group. Eight reported a history of domestic violence, seven histories of sexual abuse, five childhood sexual abuse, and eight intimate partner violence (IPV).

**Conclusion:**

A post-disaster integrated GP and mental health program in a rural community can assist in identifying individuals experiencing post-disaster psychological distress using opportunistic psychological screening. The findings indicate that collaborative mental health programs may effectively support rural communities post-disaster.

## Specific Event Identifiers:

- Event: Evaluated psychological response to flood events.
- Onset Date: Flood occurred on February 2, 2012.
- Location of the Event: Southwest Queensland.
- Geographic Coordinates: Latitude: -26.4830, Longitude: 147.9660, Elevation: 336.3 m (1,103 ft), Area: 676.4 km2 (261.2 sq mi).
- Dates of Observations Reported: Program commenced week of March 26, 2012. Program ceased week of June 4, 2012.Program commenced week of March 26, 2012. Program ceased week of June 4, 2012.
- Response Type: Program provided psychological assessment and intervention for those affected by the flood.
- Addendum: Note as the town has a small population, disclosure of the town’s name risks identification of people who live or lived in the region.

## Introduction

In 2021, Eastern and Central Australia experienced the heaviest recorded rainfall since 1900. The rain event resulted in extensive flooding across large areas of Queensland and New South Wales (NSW).^1^ Persistence of a La Niña event through the 2020-2021 summer produced further heavy rainfall and flooding. Some regions of southeast Queensland and NSW subsequently experienced multiple separate flood events.^2^ Disaster declarations were made for three events in Queensland to initiate joint State and Commonwealth Disaster Recovery Funding Arrangements (DRFA). Similar storm and flood disaster declarations were made for various locations within NSW from July 2021 through March 2022.^3^ The flooding was linked to the death of 23 people during the summer events of 2021-2022. Housing, business, and infrastructure damage was estimated to cost $2.5 billion.^4^

In addition to the grief and the adverse immediate and long-term psychological consequences of the floods, many experienced personal losses such as housing and possessions. There was wide-spread damage to education facilities, transport, and public utility infrastructure and increased demands on clinical and public health services in affected regional and rural centers.

From a psychosocial perspective, community supports that include the non-government sector, primary health care, and specialized mental health services are key elements of the psychosocial response to natural disasters. While recognized as a vital aspect of the disaster response, general practitioners (GPs) remain under-utilized and under-recognized in disaster preparedness and recovery systems. While actively involved in monitoring their patient’s pre-existing illnesses, GPs are also engaged in the long-term monitoring of patients post-disaster. They also have an important role in early intervention programs that identify the emergence of new physical and mental health illnesses.^5^ Successful early intervention strategies require the integration of GPs into the broader disaster health response structure. This integrated approach can enhance the health system’s resilience and maximize its response capacity through communication and early intervention.^6–8^

Strategies to increase integration of the health system and GPs continue to be challenged by the nature and magnitude of disasters, the geography of affected regions, resource availability pre- and post-disaster, and pre-existing barriers between public health providers, including mental health services and GPs.^9–13^

Responding to a disaster will also require an understanding that the psychosocial impacts vary according to age, gender, and the regional environment. Evidence indicates the education system offers an appropriate point of access to identify at-risk children and families and provide evidence-based interventions for the child/adolescent and the family post-disaster.^14,15^ The most likely points of service access for older adults are GPs or emergency departments. “Help-seeking behavior” for those aged 18 through 65 is influenced by factors such as the community and work environment and the need to support their family. Consequently, they may delay or avoid contact with health providers^16–20^ with the likelihood of under-utilization of available treatment resources.^21–27^

The flooding across Queensland and NSW in 2021-2022 highlights the tests governments at all levels experience during natural disasters. The repeated damage to public and private infrastructure, multiple episodes of injury and loss of life, and the expanding demands for psychosocial services in affected communities challenged all involved in the disaster response. This paper reports on a strategy that sought to address the psychosocial needs of a region affected by three major flood events that caused significant property damage and evacuations. The floods occurred from March 2010 through February 2012.

A mental health clinician (MHC) funded through the Queensland Mental Health Natural Disaster Recovery Plan 2011-2013 (QMHNDRP)^28,29^ was placed within the region’s rural primary care service. Through opportunistic screening of patients attending the GP practice, the program aimed to identify those with unmet mental health needs and to facilitate their access to local mental health care. The program added to the already established 24-hour state-wide access line, psychological first aid program, NGO community-based supports, and specialist mental health programs/SMHPs established following the 2010-2011 natural disasters.^28,30,31^ The GP-referred service commenced following feedback from clinicians and in discussion with the local GP and public health team. The MHC placement was two days per week for ten weeks, beginning eight weeks after the flood.

## Method

The GP’s receptionist used a standardized script when patients attended the practice. Each person was asked, “Were you personally affected by the floods?” If they answered “Yes,” the receptionist advised, “the Doctor is interested in knowing how things are for you since the floods; would you mind answering a few questions and handing your answers to the doctor when you see him?” The receptionist provided the individual with the questionnaires (Primary Care Posttraumatic Stress Disorder [PTSD] Scale [PC-PTSD]^32^ and Kessler 6 [K6]^33,34^), and when completed, these were handed to the doctor.

The GP scored the patient’s answers using a template and interpretation sheet. Should the individual’s PC-PTSD score fall within a designated clinical range (higher than two), the GP suggested the individual meet with the MHC, who was described as a disaster trauma specialist. Individuals who scored two or below were provided information about coping with a natural disaster.

### Measures

Probable PTSD was measured using the PC-PTSD. The measure was developed as a screening tool for the US Department of Veterans Affairs (Washington, DC USA). Subsequent studies in the primary care setting indicate solid psychometric properties, similar to more extensive screening measures. An optimal cut-off score of higher than two classifies 83% of patients correctly, with sensitivity and specificity values of 85% and 82%, respectively.^32,35^

The K6 is a subset of the K10, using Items 2, 4, 5, 8, 9, and 10, with total scores ranging from six to thirty. Studies indicate the K6 is only marginally less sensitive and specific when compared to the K10, with one study finding no statistically significant difference between the two measures when screening for a mental disorder. Cut-off scores in different studies have varied between 13 and 14.^33,36^

Patients participating in the program were advised of the outcome of the assessments and that the evaluations and clinical notes would be entered into their GP record. Clinical content was also entered into the state-wide electronic mental health record. Patient data were deidentified and collected as part of the program evaluation and independently analyzed. No data were collected from those who did not seek treatment.

### Ethics Approval

HREC/14/QPAH/472 (Metro South Health Ethics Committee; Queensland, Australia) – A retrospective evaluation of the outcomes of state-wide disaster mental health programs established and delivered following the Cyclones and Floods of 2010-2011.

## Results and Data Analysis

Due to the small number who participated in the study, a descriptive analysis was, in the main, utilized. The number who declined the assessment was not recorded. Sixty screening assessments were completed. Fourteen patients scored higher than two on the PC-PTSD and were referred to the MHC by the GP. All the screened patients completed the K6 as a secondary screening measure. Those not scoring higher than two on the PC-PTSD scale were not referred for further assessment. As per protocol, those who did not exceed the PC-PTSD cut-off score received from the GP information on coping after a natural disaster, the availability of support services, and advice on how to contact the GP.

The average age of the 14 patients referred for further assessment was 53 (34-71) years. The referred group PC-PTSD mean score was 3.14 and K6 mean score was 19 (13-26). In contrast, those not referred to the MHC had a PC-PTSD mean score of 0.72 and K6 mean = 7.30. As would be expected due to the selection process, the treatment and non-treatment groups differed significantly (PC-PTSD: *t*12.60758; P <.00001 and K6: *t*15.18068; P <.00001). The majority were female (n = 11; Table 1). All those in the treatment group experienced property damage that required relocation to other accommodation (Table 2). Of the 14 patients, eleven owned their homes, four were employed, and ten described previous exposure to natural disasters.

**Table 1.** Data PC-PTSD and K6, Age and Gender

Test	Mean	Range	P Value
PC-PTSD Mean Score (Treatment Group)	3.14		P <.00001
PC-PTSD Mean Score (Non-Treatment Group)	0.72		
K6 Score for the Total Group	10	6-26	
K6 Score Treatment Group	19	13-26	P <.00001
K6 Score Non-Treatment Group	7.30	6-11	
Age	53	34-71	
**Gender**	Female (11), Male (3)		

Abbreviations: PC-PTSD, Primary Care Posttraumatic Stress Disorder [PTSD] Scale; K6, Kessler 6.

**Table 2.** Personal History/Demographics

Descriptor	Experience YES	Experience NO
Identify as Aboriginal or Torres Strait Islander	3	11
Own Home	11	3 (rental)
Married	7	7 (s-2, w-1, d-4)
Employed	4	11 (social security)
Property Damage/Relocation	14	nil
Health Worse	14	nil
Relationship Changes	14	nil
Increase Use of Alcohol, Drugs, and Tobacco	8 (alcohol -1, drugs-1, tobacco-6)	6
Family Hx Mental Illness	4	10
Family Hx Domestic Violence	8	6
Family Hx Alcohol/Drugs	10	4
Previous Disaster Exposure	9	5
Hx Childhood Abuse	5	9
Hx Sexual Abuse	2	12
Hx Domestic Violence	8	6
Hx Mental Illness	1	13
Complicated Bereavement	7	7
Suicidal Ideations/Attempt	2	12

Abbreviation: Hx, history.

**Figure 1.**
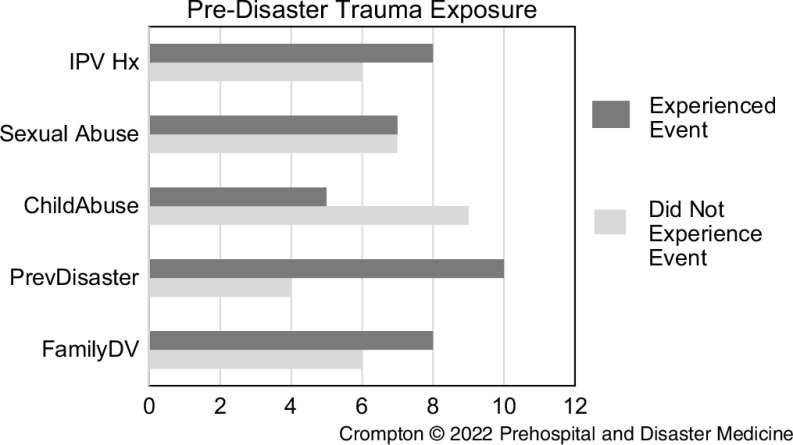
Trauma Experience of the Referred Group. Abbreviations: IPV, intimate partner violence; Hx, history; FamilyDV, family domestic violence.

A history of previous trauma exposure was notable in the intervention group. Eight reported a developmental history of exposure to domestic violence, with seven describing a history of sexual abuse and five childhood sexual abuse. Eight of the 11 women reported a history of intimate partner violence (IPV); Figure 1.

**Figure 2.**
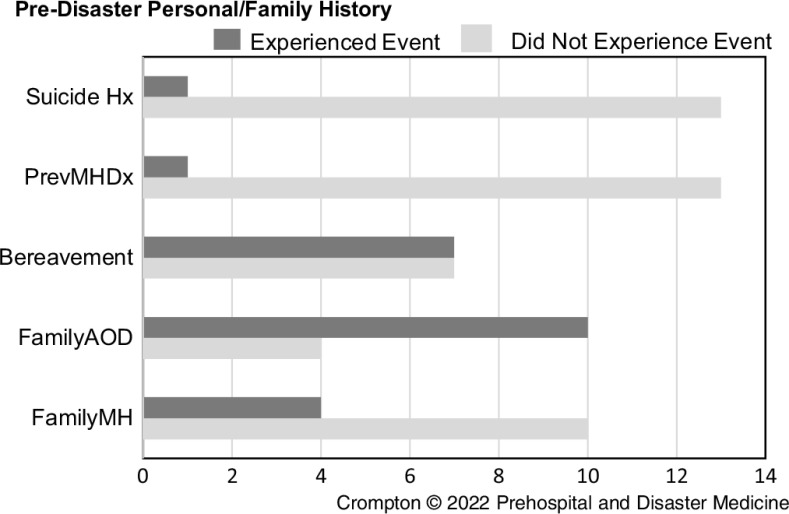
Pre-Disaster Personal and Family History. Abbreviations: Hx, history; PrevMHDx, previous mental health diagnosis; FamilyAOD, family history of alcohol and/or drug disorder; FamilyMH, family history mental illness.

One person reported a previous history of mental illness. Ten patients described a family history of drug use and/or excess alcohol consumption, and four had a family history of mental illness. Seven of the referred group recounted an abnormal bereavement reaction, with one describing a history of a suicide attempt (Figure 2).

Trauma-Focused Cognitive Behavior Therapy (TF-CBT) was provided to five patients. The mean number of treatment sessions was 5.86; the remaining nine declined TF-CBT. Psychoeducation, self-help programs (eg, stress management), or cognitive and behavioral interventions were provided to those who declined TF-CBT.

One individual was identified as suicidal and referred to an MHC trained in suicide prevention.

## Discussion

This paper presents a case study of one practice in one rural community. The importance of collaboration, engagement, and screening is reflected in the role of the GP’s administration staff and the number of patients who agreed to participate (n = 60). Almost 20% were identified as at-risk for PTSD (PC-PTSD higher than two). Notable was the number with a history of previous trauma exposure either due to a natural disaster or the experience of family trauma (Figure 1). Exposure to traumatic events is a common occurrence, with studies indicating seven out of ten people world-wide describe exposure to one or more events in their lifetime. For most people, the outcome is a return to their pre-event level of function. Studies also reported post-traumatic growth following military and civilian trauma.^37–39^

A traumatic experience is a *sine qua non* for developing PTSD and comorbid psychopathology. However, there is apparent heterogeneity concerning the outcome of trauma exposure with gender, the nature of the event, age of trauma occurrence, past mental health history, and previous trauma exposure influencing the psychosocial outcomes. A Norwegian study found females, compared to males, are more likely to be exposed to sexual abuse (P = .011), rape (P <.001), and IPV (P <.017); physical violence (P <.001); or observe traumatic events (P = .014). One potential outcome is PTSD, with males more likely to attempt self-medication using alcohol, while for others, the outcome is reflected in altered interpersonal relationships. The findings reflect previous studies that have identified a link between the effect of trauma exposure and multiple risk factors such as developmental history and social and economic factors.^40,41^

The data derived from this small cohort group mirror the findings of more extensive studies; females more likely to seek assistance, the influence of socio-economic factors (over 75% receiving social security), and for many, the likelihood of non-disclosure of a personal history of trauma exposure. The treatment group, in comparison to the findings of an Australian epidemiological survey, were more likely to have been exposed to natural disasters (70.0% versus 19.9% [males] and 12.7% [females]), and similarly, many experienced interpersonal trauma such as IPV or sexual abuse.^35,42,43^ One-third reported childhood sexual abuse (Figure 2). Similar to other studies, engagement in treatment was challenging^35,43^ with only one-third participating in the TF-CBT and none attending for ten sessions.

The descriptive data from this study reinforce and emphasize the need for GP skills in screening and identifying risk factors to address their patients’ unmet mental health needs and the increased risk of adverse psychosocial outcomes following a traumatic experience.^44,45^ Interestingly, the patient group did not report a history of mental illness diagnosed by a clinician. A family history of substance use was reported by 70% of the group, and 50% described symptoms consistent with a history of a complicated bereavement reaction. Anecdotally, many referred to the MHC had neither reported nor discussed their trauma history with their GP. Similarly, the patient with a history of a suicide attempt and now experiencing suicidal ideation only identified this aspect to the MHC after entering the program.

Although it could be argued that this group of patients may not reflect the broader community, recognition of mental illness symptoms and avoidance or accessing mental health care is not uncommon in rural communities.^46–48^ Factors influencing mental health care access in rural communities include stigma and stoicism with the idea it is a person’s task to find a way through the problem. Other issues impacting “help-seeking” for mental health care compared to physical health care include the availability of trained health professionals, concerns regarding confidentiality, and gender bias with men less likely to seek assistance than women.^49–52^

When considering patient engagement with the program, there is a need to reflect on the many influences on patient decision making concerning treatment participation. In the case of rural people, there are potential issues of trust related to the MHC that is seen as external to the community, or the program messaging does not connect with the people. A systematic review by Ferris-Day, et al also identified the need for strategies that support help-seeking, such as gender-specific strategies, mental health literacy programs, and the development of support networks to enhance all health outcomes.^46^ Barraclough, et al highlighted the importance of integrating mental health services with primary care and not-for-profit organizations in rural communities.^52^

## Limitations

The study is limited by several factors, including the number screened and the small size of the treatment group, the absence of Gold Standard assessment instruments, such as the Clinician-Administered PTSD Scale (CAPS-5), the Structured Clinical Interview for the DSM (SCID-5), assessments of substance use and global function, for example, the GAF, or WHOQOL. The study does not include post-therapy evaluations, and the non-treatment group has not been evaluated in terms of past history, employment, and health status.

## Conclusion

A post-disaster integrated GP and mental health program in a rural community demonstrated that individuals suffering from the effects of a natural disaster could be identified using well-recognized screening instruments. The findings suggest that individuals at-risk may present at a GP clinic for other health reasons, and in the absence of a screening program, their underlying concerns may go unaddressed.

The GP, MHC, and patients expressed support for the program and perceived the process implemented in the primary care setting as a valuable adjunct to the services provided by the clinic.

Although limited in sample size, the results suggest a screening program is a promising primary care option following a natural disaster. The screening and intervention program augmented clinical services offered by the GP and importantly identified at-risk individuals who had not previously discussed their prejudicial developmental and current psychosocial experiences.

The elucidation of an inclusive history requires time and skill, and unless asked or screened for, psychological symptoms may remain undisclosed and unaddressed. While likely to present to a primary care setting for problems other than psychological distress, at-risk individuals can present with symptoms that mask the underlying distress, risking a delay in treatment. The lost intervention opportunity may be associated with adverse psychological outcomes and impact employment and relationships.
